# Peyronie's Disease: Evaluation and Review of Nonsurgical Therapy

**DOI:** 10.1100/tsw.2009.92

**Published:** 2009-07-27

**Authors:** Michael R. Abern, Laurence A. Levine

**Affiliations:** Department of Urology, Rush University Medical Center, Chicago, USA

**Keywords:** Peyronie’s disease, penile induration

## Abstract

The purpose of our study was to outline the evaluation of the Peyronie's disease (PD) patient and review the available nonsurgical treatments. A review of the literature on oral, intralesional, external energy, iontophoresis, and mechanical therapies for PD was performed. PubMed was utilized to find all published articles, and several meeting abstracts were reviewed for data ahead of publication. Our medical evaluation of the PD patient is described. The published results of available treatment options are reviewed, with recommendation by the authors for appropriate nonsurgical management of PD. There are no available validated questionnaires for PD, but a thorough history and focused physical examination, including measurement of erect penile deformity, will help the clinician make the diagnosis and guide treatment options. Although there are many published reports that show efficacy of nonsurgical therapies for PD, there is a lack of large-scale, multicenter, controlled clinical trials, which makes treatment recommendations difficult. Careful review of the literature does suggest that there are treatment options that make scientific sense and appear to stabilize the disease process, reduce deformity, and improve function. Offering no treatment at all will encourage our patients to pursue alternative treatments that may do harm, and misses the opportunity to do some good. Clearly, further work is necessary to develop safe and effective nonsurgical treatments for PD.

## DEFINITION

Peyronie's disease (PD) is a psychologically and physically devastating disorder that is manifest by a fibrous, inelastic scar of the tunica albuginea, resulting in a palpable penile scar in the flaccid condition and causing penile deformity, including penile curvature, hinging, narrowing, shortening, and painful erections. In spite of multiple treatment options offered since Francois de la Peyronie described PD in 1743[1], this disorder remains a considerable therapeutic dilemma even to today's practicing physicians.

## EVALUATION OF THE PD PATIENT

Thorough evaluation of the PD patient is essential, not only to diagnose the disease correctly, but also to guide treatment. Currently, no universally accepted standardized evaluation for the PD man exists, nor has a validated questionnaire been developed. A suggested guideline for initial evaluation of the PD patient, including history, physical exam, and imaging analysis, has been recommended[2] and is outlined below.

The subjective assessment begins with the patient interview. The history should be focused on the onset and duration of symptoms, the patient's presenting signs and symptoms, and the presence or absence of pain. It is particularly useful to elucidate whether the patient continues to experience pain at the time of the initial evaluation, as this may represent a man in the acute phase of the disease. Pain may be present with palpation, erection, or during coitus, and should be differentiated, as this may indicate a different etiology or degree of acute inflammation. The patient's subjective curvature deformity should be noted. It should also be noted that up to 90% of men with PD may present with diminished rigidity. In our experience, up to 50% of men with erectile dysfunction (ED) and PD have reported the onset of ED preceding PD. It is also important to know what, if any, prior PD therapies the patient has undergone, as this may help to guide future treatment.

A detailed past medical and sexual history should be part of the initial evaluation of every man with PD. The medical history should focus on personal or family history of wound-healing disorders, including Dupuytren's contracture or Lederhose disease, which is reported in up to 20% of patients with PD. Any risk factors for ED, such as dyslipidemia, hypertension, atherosclerotic disease, history of tobacco use, and diabetes, should be queried. The patient's baseline erectile function should be assessed using a validated questionnaire. Although a validated PD questionnaire is still in development, the IIEF-EF may be used to gauge the patient's baseline sexual function.

The objective evaluation begins with the physical exam. Although the focus should be on the genital exam, an examination of the hands and feet is appropriate given the patient's history. Measurement of penile length is critical, as the loss of penile length is not only a known complication of PD, but is also a source of great concern among patients. The penis should be measured stretched in its flaccid state, and stretched penile length (SPL) should be measured dorsally from pubis to corona or meatus. Note that the suprapubic fat pad should be compressed during measurement. Objective evaluation of curvature is best performed using penile duplex ultrasound after pharmacologic stimulation to produce a full erection equal to or better than the patient's erection at home. Simple erection induction in the office will allow objective assessment of deformity. Duplex ultrasound will allow assessment of vascular flow rates, the degree of curvature as measured with a goniometer or protractor, the presence and location of PD plaque(s), and the presence of any hinge effect. In addition, plaque calcification can be assessed. Autophotography should not be used as the sole means for curvature measurement, as this modality can be inconsistent and inaccurate.

The final portion of the PD evaluation is objective assessment of the patient's erectile capacity and penile sensation. During duplex ultrasound, the patient should be asked to grade his pharmacologic erection as compared to home erections. Although little exists in the PD literature, penile somatosensory testing has shown value in the ED population and is correlated with IIEF scores[3]. We perform biothesiometry as a noninvasive, office-based method to assess the baseline neurological status of the penis. Using the distal phalanx of the index fingers as positive control and the ventral surface of bilateral thighs as negative control, the point at which vibratory sensation is appreciated should be measured on the mid-shaft bilaterally and on the glans. This threshold may contain prognostic value for patients experiencing transient penile sensory complaints after therapy.

## MONITORING TREATMENT EFFICACY

It is important to monitor both the subjective and objective response to any PD treatment. Although there is no standard subjective questionnaire, some effort should be made to assess the patient's perception of changes in penile length, girth, curvature, erections, and pain. Objective change in length can be monitored with SPL. We repeat a duplex ultrasound with pharmacologic stimulation at the conclusion of a nonsurgical management protocol in order to measure the changes in erect curvature and plaque characteristics.

## NONSURGICAL THERAPY FOR PEYRONIE'S DISEASE

Since the first description of PD in the literature, physicians have been searching for medical therapy options with little confirmed success. Consistent successful medical therapies continue to evade the practicing urologist, although current research into the molecular pathophysiology of PD may one day lead to a medical cure. Several nonsurgical options, however, are currently available and may stabilize or reduce deformity and improve sexual function. The evaluation of their efficacy has been compromised by small clinical trials and without, in most cases, any placebo control. Outcomes are difficult to interpret with an absence of a validated questionnaire, and in a disease in which spontaneous improvement has been noted in 5–12% of patients[4,5,6,7]. Below, the nonsurgical options for treatment of the pain and curvature of PD, including oral, topical, intralesional, external energy, mechanical, and combination therapies, are presented.

### Oral Therapies

- **Vitamin E** — Vitamin E was the first oral therapy to be described for the treatment of PD[8]. Vitamin E is a fat-soluble vitamin that is metabolized in the liver, excreted in bile, and is thought to have antioxidant properties in humans. Oxidative stress and the production of reactive oxygen species (ROS) are known to be increased during the acute and proliferative phases of wound healing, as it is neutrophils and macrophages that produce these ROS species[9], and the inflammatory phase of wound healing has been shown to be prolonged in Peyronie's patients[10]. Thus, a rationale for vitamin E use is that it may decrease the oxidative damage to the tunica. Gelbard et al.[6] compared vitamin E therapy to the natural history of PD in 86 patients; no significant differences were found between the two groups in terms of curvature, pain, or the ability to have intercourse. In 1983, Pryor and Farrell performed a double-blind, placebo-controlled, cross-over study evaluating vitamin E for the treatment of PD in 40 patients[11]. No significant improvements were noted in plaque size or penile curvature. More recently in 2007, Safarinejad et al.[12] published a double-blind, randomized trial of vitamin E with or without proprionyl-L-carnitine (PLC) for treatment of early PD. Patients were randomly assigned to receive either vitamin E, PLC, vitamin E and PLC, or placebo. No significant improvement in pain, curvature, or plaque size was noted in any treatment group as compared to placebo. In the opinion of the authors, vitamin E is not recommended for the treatment of PD, as there is no evidence of benefit in placebo-controlled trials.
- **Colchicine** — Colchicine is an antigout medication that inhibits fibrosis and collagen deposition primarily through its inhibition of the inflammatory response through inhibition of neutrophil microtubules[13]. Colchicine has been used both as primary oral therapy for PD as well as in combination with others. Akkus et al.[14] administered an escalating dose of colchicine in a nonrandomized, nonplacebo-controlled fashion to 19 patients with PD over a 3–5 month period. Thirty-six percent of the patients noted a reduction in curvature and 63% noted an improvement in the palpable plaque. Seventy-eight percent of the patients that were experiencing painful erections at the time of treatment initiation had resolution of this symptom. Kadioglu et al.[15] treated 60 patients with PD using 1 mg of colchicine twice daily, with a mean follow up of 11 months. They found significant improvement of pain in 95% of the men; however, 30% of the patients reported improved curvature, while 22% of patients reported worsened curvature. Safarinejad performed a randomized, placebo-controlled trial of colchicine in 2004 with 84 men[16]. It was found that colchicine is no better than placebo for improvement of pain, curvature angle, or plaque size as measured by ultrasound. Colchicine is not recommended by the authors due to its lack of demonstrated efficacy in placebo-controlled trials. The agent is also associated with gastrointestinal distress, including diarrhea, and with rare, aplastic anemia.

- **Potassium aminobenzoate** — Potassium aminobenzoate (Potaba, Glenwood) is a member of the vitamin B complex that is believed to increase the activity of monoamine oxidase in tissues, thereby decreasing local levels of serotonin and thus possibly decreasing fibrogenesis. Potassium aminobenzoate is used for other conditions, including scleroderma, dermatomyositis, and pemphigus. Zarafonetis and Horrax[17] first described the use of potassium aminobenzoate for the treatment of PD, and a subsequent European study published in 1978 reported a 57% improvement rate with 9% complete resolution in a pooled cohort of 2,653 patients[18]. This study, however, did not include a control or placebo group. In 1999, Weidner et al.[19] published a randomized, placebo-controlled trial of potassium aminobenzoate given 3 g orally four times per day for 1 year in 103 men. The only significant difference found between the two groups was plaque size, which was not and has not been shown to correlate with a decrease in penile curvature. A 2005 follow-up study, also by Weidner et al.[20], suggested that the use of potassium aminobenzoate may protect against progression of PD plaques. Potassium aminobenzoate is expensive and has low tolerability due to gastrointestinal side effects. It is also not recommended by the authors due to a lack of evidence regarding its efficacy in the treatment of PD.
- **Tamoxifen citrate** — Tamoxifen is a nonsteroidal antiestrogen that acts by competing with estrogen binding sites in target tissues. In addition, tamoxifen affects the release of TGF-β from fibroblasts, and blocks TGF-β receptors, thus potentially reducing fibrogenesis[21,22]. In 1992, Ralph et al.[21] investigated tamoxifen in 36 patients with recent-onset PD (duration less than 4 months). Eighty percent of the patients reported a reduction in pain, 35% reported a subjective reduction in curvature, and 34% reported a decrease in plaque size. A follow-up study in 1999 by Teloken et al.[23] failed to show any statistically significant difference between tamoxifen and placebo, and there was a reported increase of alopecia in the active treatment group. We do not recommend the use of tamoxifen.
- **Carnitine** — Carnitine is a naturally occurring metabolic intermediate. Carnitine facilitates the entry of long-chain fatty acids into muscle mitochondria, which are then used as energy substrate. Carnitine is also thought to inhibit acetyl coenzyme-A, which may aid in the repair of damaged cells. Biagiotti and Cavallini examined the use of carnitine for PD in 2001[24]. Forty-eight men were divided into two groups to receive either tamoxifen at 20 mg twice daily for 3 months or acetyl-L-carnitine 1 g twice daily for 3 months. Overall, the men taking carnitine saw greater improvement in curvature and had statistically significant improvement in pain. In addition, the patients taking carnitine reported far fewer side effects as compared to tamoxifen. Based on the results of the PLC arm of the afore-mentioned, placebo-controlled trial by Safarenijad et al., however, we do not recommend carnitine therapy.
- **L-Arginine** — L-Arginine is an amino acid that, when catalyzed by nitric oxide synthase (NOS), combines with oxygen to ultimately form nitric oxide (NO). It is known that inducible NOS (iNOS) is expressed in the fibrotic plaques of PD and that long-term suppression of iNOS exacerbates tissue fibrosis[25]. In 2003, Valente et al. reported that L-arginine given daily in the drinking water of rats that were injected with TGF-β1 to induce PD plaques resulted in an 80–95% reduction in plaque size and in the collagen/fibroblast ratio[25]. In addition, L-arginine was found to be antifibrotic *in vitro*. This suggests that L-arginine, as a biochemical precursor of NO, may be effective in reducing PD plaque size. The authors offer L-arginine 1000 mg twice daily to our patients in combination with other therapies, but clearly, further human trials are needed before this agent can be strongly recommended.
- **Pentoxifylline** — Pentoxifylline is a nonspecific phosphodiesterase (PDE) inhibitor. Valente et al.[25] found that normal human and rat tunica albuginea, as well as PD plaque tissue, express

PDE5A-3 and PDE4A, B, and D. In their *in vitro* study, PD fibroblasts were cultured with pentoxifylline and were found to have increased cAMP levels and reduced collagen I levels as compared to controls. In addition, pentoxifylline given orally to a TGF-β1–induced PD rat model resulted in a decrease in PD plaque size and collagen/fibroblast ratio. Brant et al. reported a single case report of successful PD treatment using pentoxifylline alone[26]. Further studies are required to examine pentoxifylline definitively for the treatment of PD; however, its known biochemical effect and early animal-model success make it an attractive option for oral therapy.

## Topical Therapies

- **Verapamil** — Interest in topical verapamil for the treatment of PD followed its success as an intralesional agent (see below). However, one study demonstrated that tunica albuginea tissue concentrations of verapamil are not achievable through topical application[27]. A recent, three-arm trial without a known placebo demonstrated benefit with topical verapamil[28], but this study was significantly compromised[29]. Thus, the use of verapamil as a topical agent for PD is not recommended.

## Intralesional Therapies

- **Steroids** — The powerful anti-inflammatory effect of steroids made them early-investigated agents for intralesional therapy of PD. In 1954, Bodner et al.[30] reported improvement in 17 patients treated with intralesional hydrocortisone and cortisone. In 1975, Winter and Khanna[31] showed no difference between patients treated with dexamethasone injections and the natural history of the disease. In 1980, Williams and Green[32] published a prospective study using intralesional triamcinolone. All patients were observed for 1 year after study enrollment; during that time, only 3% of the patients reported improvement. Triamcinolone was administered every 6 weeks for 36 weeks; 33% of the patients reported subjective improvement, particularly in pain and plaque size. Currently, the use of intralesional steroids is discouraged due to the side effects of local tissue atrophy, fibrosis, immune suppression, and lack of objective measures of benefit.
- **Collagenase** — Collagenase was first studied *in vitro* by Gelbard et al. in 1982[33]. A subsequent clinical trial by that group[34] demonstrated subjective improvement in 64% of the patients within 4 weeks of treatment. A decade after their initial study, they published their findings of a double-blind trial in 49 men[35]. Statistically significant improvement in curvature was noted in the collagenase-treated group; however, maximal improvement ranged from 15–20$^o$ and was only seen in the patients with curvatures of less than 30$^o$ and plaques of less than 2 cm in length. A prospective, single-center trial was performed by Jordan that showed significant benefit in curvature and plaque width with intralesional, clostridial, collagenase injections[69]. Multicenter, controlled trials of intralesional collagenase are currently in progress.
- **Verapamil** — Verapamil is a calcium channel blocker that has been shown in *in vitro* studies to inhibit local extracellular matrix production by fibroblasts, to reduce fibroblast proliferation, to increase local collagenase activity, and to affect the cytokine milieu of fibroblasts[36,37]. In 1994, Levine et al.[38] reported on 14 men who underwent a dose-escalation trial of biweekly intralesional injections of verapamil for 6 months. Significant improvement in plaque-associated narrowing was noted in all patients and curvature was improved in 42%. The first randomized, single-blind trial of intralesional verapamil was published in 1998[39]. Significant differences were noted in terms of erection quality and plaque volume. A trend towards improvement in curvature was also noted. As a follow-up, Levine and Estrada reported on 156 men enrolled in a prospective, nonrandomized trial of PD men with a mean follow-up of 30.4 months[40]. A local penile block was performed with 10–20 ml 0.5% bupivicaine, followed by injection of 10 mg verapamil diluted in 6 ml sterile normal saline (total volume 10 ml) into the PD plaque using one to five skin punctures, but with multiple passes through the plaque. The goal was to leave the drug in the needle tracks, not to tear or disrupt the plaque. Injections were administered every 2 weeks for a total of 12 injections. Eighty-four percent of the patients with pain achieved complete resolution, 62% were found on objective measurement to have improved curvature ranging from 5–75$^o$ (mean 30$^o$), and only 8% of the patients had measured worsening of curvature. More recently, Bennett et al.[41] administered six intralesional injections (10 mg in 5 ml) every 2 weeks to 94 consecutive patients with PD. Follow-up was at 5.2 months after completion of the sixth injection. Eighteen percent of the patients (n = 17) were found to have improved curvatures (average improvement 12$^o$), 60% (n = 56) had stable curvature, and 22% (n = 21) had increased curvature (average increase 22$^o$). All patients with pretreatment penile pain had improvement at follow-up. The authors suggest that these data support intralesional verapamil for the stabilization of PD. It may be that six injections provide stabilization, but are insufficient to accomplish reduction of curvature. Currently, we recommend a trial of six injections with each injection occurring every 2 weeks. If no improvement is noted by the patient, the therapy may be terminated, the verapamil dose can be increased to 20 mg, or interferon (IFN) injections may be offered. We consider verapamil unlikely to benefit patients with ventral plaques or extensive plaque calcification. We recommend a course of intralesional verapamil based on the published data, and low cost and favorable side-effect profile.

- **Interferons** — In 1991, Duncan et al.[42] reported that IFNs decrease the rate of proliferation of fibroblasts in Peyronie's plaques *in vitro*, reduce the production of extracellular collagen, and increase the activity of collagenase. Initial studies performed by Wegner et al.[43,44] demonstrated low rates of improvement, but a high incidence of side effects, including myalgias and fever. In 1999, Ahuja et al.[45] reported on 20 men who received $1 \times 10^6$ units of IFN-α-2b biweekly for 6 months. One hundred percent of the patients reported softening of plaque, 90% of the men presenting with pain had improvement, and 55% had a subjective reduction in plaque size. Dang et al.[46] administered $2 \times 10^6$ units to 21 men biweekly for 6 weeks, and found objective curvature improvements in 67% and improvement in pain in 80%. Seventy-one percent of the patients reported improvement in ED symptoms. In 2006, Hellstrom et al.[47] reported on a placebo-controlled, multicenter trial of 117 patients who underwent biweekly injections of $5 \times 10^6$ units for a total of 12 weeks. Average curvature in the treatment group improved 13 vs. 4$^o$ in the placebo arm, and 27% of the patients in the treatment group had measured improvement vs. 9% of the saline group. Pain resolution was noted in 67% of the treatment patients vs. 28% for placebo. A significant contribution made by this study was to discount the theory that the needle passes provide therapeutic benefit, as the placebo arm showed very little change in curvature. The published results for IFN therapy are encouraging, but require further investigation to adequately determine efficacy, dosing regimens, and side effect profiles before its routine use in PD patients.

## External Energy Therapies

- **Penile electroshock wave therapy (ESWT)** — Local penile ESWT has been suggested to be helpful. Various hypotheses about its mechanism of action exist, including direct damage to the plaque resulting in an inflammatory reaction with increased macrophage reaction leading to plaque lysis, improved vascularity resulting in plaque resorption, and the creation of contralateral scarring of the penis resulting in "false" straightening[48]. Hauck et al.[49] randomized 43 men to ESWT or oral placebo for 6 months. No significant effect was noted in terms of curvature, plaque size, or subjective improvement in sexual function or rigidity. More recent work from a German group[50,51] randomized 102 men to ESWT or placebo shocks. There was no statistically significant difference found between the groups for plaque size, improvement of deformity, or sexual function post-treatment. ESWT currently is not recommended as therapy for PD.

- **Iontophoresis** — Iontophoresis involves the transport of ions through tissue by means of an electric current. Several studies have investigated the efficacy of topically applied verapamil with or without dexamethasone with enhanced penetration using iontophoresis[52,53,54,55]. In 2002, Levine et al. confirmed that verapamil was found within the exposed tunica albuginea by examining surgically retrieved tunica albuginea from patients after a single intraoperative exposure during plaque incision and grafting surgery[56]. Di Stasi et al. recently reported on a prospective, randomized study of 96 patients treated with 5 mg verapamil plus 8 mg dexamethasone using iontophoresis vs. 2% lidocaine delivered electromotively[55]. Forty-three percent of the patients in the verapamil/dexamethasone group noted objective improvement in plaque size and curvature; no changes were noted in the lidocaine group. In 2005, Greenfield et al.[57] reported on the use of 10 mg verapamil vs. saline iontophoresis. Patients were assessed using papavarine-induced erections prior to and 1 month after treatment. Sixty-five percent of the patients in the verapamil group demonstrated improvement in curvature vs. 58% in the saline group. Mean curvature improvement was 9.1$^o$ in the treatment group vs. 7.6$^o$ in the saline group, which is clearly not as robust a response as reported with intralesional verapamil injections. The authors suggested that the electric current itself may have some beneficial effect on wound healing, which is known and supported in the dermatologic literature[58]. We offer home iontophoresis using verapamil and dexamethasone as second-line therapy for men who cannot participate in an intralesional verapamil injection program.

## Mechanical Therapies

- **Vacuum devices** — Vacuum devices have been used extensively to treat ED and for postprostatectomy penile rehabilitation. Theoretically, a vacuum device will provide stretching force to the tunica albuginea that may aid in remodeling of PD plaques. We recently evaluated the addition of a vacuum device (SOMA [Augusta Medical Systems LLC, Augusta, GA]) to our existing intralesional verapamil protocol in 65 men and found no significant improvement with regard to measured penile deformity or patient-reported subjective outcomes[59]. We feel that the vacuum device provides inadequate forces to the tunica and is no longer part of our nonsurgical management strategy for PD.
- **Penile traction devices** — The use of tissue expanders has long been a mainstay of treatment in the orthopedic, oral-maxillofacial, and plastic surgical fields. It is well documented that gradual expansion of tissue results in cellular proliferation via several mechanisms, including cyclin D1–mediated cell cycle proliferation[60], paracrine signaling of FGF and PDGF[61], and activation of mechanosensitive calcium channels and the IP3/DAG pathway[62]. Recently, initial work has been done to evaluate the efficacy of the Penis Extender device (FastSize LLC, Aliso Viejo, CA) for the treatment of PD. A pilot study at Rush University Medical Center of 10 patients found that daily application of the device for 2–8 h/day for 6 months resulted in a 33% measured improvement in curvature (ranging from 10 to 45$^o$ improvement and resulting in a reduction in mean curvature from 51 to 34$^o$), an increase in flaccid stretched penile length ranging from 0.5–2.0 cm, and an improvement in hinge effect in all those with advanced narrowing or indentation[63]. No patients noted recurrence or worsening of curvature during 6 months of follow-up, and there was no incidence of local skin changes, ulceration, loss of sensation, or worsening of curvature. Traction therapy is currently part of our nonsurgical management strategy (see combination therapy section below).

## Combination Therapy

- **Vitamin E and colchicine** — A placebo-controlled study by Preito Castro et al.[64] randomized 45 patients to receive vitamin E and colchicine or ibuprofen. Statistically significant improvements in curvature and plaque size were noted in the group treated with vitamin E and colchicine as compared to the group receiving ibuprofen. Patients in the vitamin E and colchicine arm reported a greater decrease in pain, although this did not reach statistical significance.

- **ESWT with intralesional verapamil injection** — In 1999, Mirone et al.[65] prospectively examined two groups of PD patients; one group was treated with ESWT, while the other received ESWT and perilesional verapamil injections. A 52% improvement in plaque size by ultrasound was noted in the ESWT-only group compared to 19% for the combination therapy. A follow-up study by the same investigators involving 481 patients demonstrated a 49% improvement in plaque size among those treated with combination therapy[66].
- **Intralesional verapamil with oral carnitine or tamoxifen** — In 2002, Cavallini et al.[67] randomized 60 men to receive intralesional verapamil plus oral carnitine or intralesional verapamil plus oral tamoxifen. Statistically significant subjective improvements in curvature, plaque size, and erectile function were found in the carnitine group. No difference in improvement of pain was found between the two groups.
- **Intralesional verapamil with traction and oral pentoxifylline and L-arginine** — We are actively studying whether the addition of traction therapy with oral pentoxifylline and L-arginine to a course of 12 intralesional verapamil injections provides any benefit. We recently presented our preliminary results in 81 men[68], which showed that the combination therapy resulted in up to a 2-cm (mean 1.2 cm) increase in penile length that was statistically significant. In addition, 59% of men on combination therapy reported a subjective curve improvement vs. 39% in the verapamil-alone group. The mean measured improvement of curvature was 21.2° in the combination group vs. 14.3° in the verapamil-alone group. The combination treatment regimen is well tolerated with only 12% of the combination therapy group dropping out of the program before its completion – none of which was due to adverse effects of the treatment. These encouraging results have established this combination of oral, intralesional, and mechanical therapy as our current nonsurgical treatment recommendation. Further study is ongoing at this time.

## CONCLUSION

PD remains a treatment dilemma, in part due to the lack of a clear understanding of its pathophysiology. Hopefully, with further basic science research and properly conducted clinical trials, novel treatments will emerge. Currently, we do not believe that oral therapy alone provides any real benefit with respect to correction of deformity, as it appears unlikely that an adequate concentration will reach the relatively hypovascular and hypocellular plaque, and placebo-controlled trials have not demonstrated benefit. On the other hand, injection of verapamil or IFN-α-2b has been shown to provide, at a minimum, stabilization of plaque/deformity progression and may improve sexual function as well. The newest noninvasive, nonsurgical treatment with prolonged traction poses a novel approach based on proven principles of tissue remodeling with encouraging preliminary results. We feel that it is possible to achieve a synergy with combination therapy using medical treatment (oral and injection), with its potential chemical effects and the mechanical effects of traction therapy. This combination may result in the best chance for a nonsurgical reduction in deformity with improvement in sexual function.
